# Health locus of control in cancer patient and oncologist decision-making: An exploratory qualitative study

**DOI:** 10.1371/journal.pone.0263086

**Published:** 2022-01-27

**Authors:** Keren Dopelt, Osnat Bashkin, Noam Asna, Nadav Davidovitch

**Affiliations:** 1 Department of Public Health, Ashkelon Academic College, Ashkelon, Israel; 2 Department of Health Policy and Management, School of Public Health, Faculty of Health Sciences, Ben-Gurion University of the Negev, Beer Sheva, Israel; 3 Oncology Institute, Ziv Medical Center, Safed, Israel; Universiteit van Amsterdam, NETHERLANDS

## Abstract

**Objective:**

To investigate how cancer patients’ and family members’ perspective and health locus of control are presented in clinical encounter decision-making.

**Methods:**

Semi-structured in-depth interviews were carried out with 16 cancer patients and 6 family members living in Israel (n = 22). Interviews were transcribed verbatim, and data were analyzed using thematic analysis.

**Results:**

Following the health locus of control model, the findings were divided into an external and internal locus of control themes, and we added a theme regarding shared decision-making. Internal locus of control sub-themes included asking for a second opinion, negotiating with the doctor, asking questions, looking for information, and fighting for their rights. External locus of control sub-themes included powerful others, oncologists, and fate. The dominant approach of most of the interviewees was an external locus of control. Women demonstrated more external locus of control than men. On the direct question of who should decide on treatment—the doctor, the patient, or both jointly—the answers ranged from only the doctor (n = 8) to together (n = 7) to only the patient (n = 8).

**Conclusions:**

This study provides insights into different aspects of locus of control in the clinical encounter involving cancer patients. The findings reflect the need to devote comprehensive attention to cancer patients’ perceptions and experiences in the clinical encounter. A patient-centered care approach and a personalized framework for decision-making in cancer care are essential to achieving better treatment outcomes. Further research can engage in the development and validation of an up-to-date health locus of control questionnaire for cancer patients based on the findings of this study.

## Background

The health locus of control (HLOC) model is derived from Rotter’s Social Learning Theory [1] and defined as “a person’s beliefs regarding where control over his/her health lies.” [2, p. 534] People with an external HLOC believe that their health is influenced mainly by others (physicians, caregivers) or by chance or fate, while people who believe that their behavior affects their health have an internal HLOC [2]. Previous studies showed HLOC influences mental health and may predict psychological adjustment and survival among cancer patients. Arraras et al. [3] found significantly lower scores of internal locus of control (LOC) among cancer patients than patients with other diseases. A passive patient role was associated with poorer adaptation than a more active role. Gibek and Sacha [4] found a negative correlation between HLOC and the duration of cancer. In addition, cancer patients are more likely than non-cancer patients to perceive others as responsible for their health, and they depend more on external sources of control, such as doctors or family members. Moreover, women had a lower internal LOC than men. HLOC also influences people’s preferences in medical decision-making and information needs [5, 6]. Patients with an internal LOC investigate and search for more information. However, patients with an external LOC allow the doctor or other "powerful" people to make decisions for them [5]. Powerful others HLOC is a belief that external individuals control one’s health [7]. Powerful others HLOC exhibited a positive and significant direct effect on trust in the physician [8].

In large Japanese study [5] examined the influence of locus of control on preferences for information and decision making, findings revealed that information preference was positively associated with decisional preference among individuals who believed their health is less dependent on powerful others.

A recent study [9] examined the information needs of cancer patients. It was found that cancer patients with a higher external locus of control used significantly more often sources of information and had more need for additional information. A significant association between a high external locus of control and complementary and alternative medicine use rate was also found among cancer patients [10].

The oncologist-patient discourse is sensitive and can be influenced by various factors, among them HLOC and levels of active vs. passive characteristics of the patient. Shared decision-making (SDM) is a beneficial practice when facing treatment decisions [11]. Physicians must discuss the treatment alternatives and their effects on the clinical outcomes and side-effects [12], and patients should actively help physicians understand their needs, values, and preferences [13]. The inclusion of cancer patients in decision-making about their treatment has received increased interest. Nevertheless, implementing strategies to the active involvement of patients in cancer care decision-making is quite complex [13, 14]. When it comes to cancer, treatment has many implications for patients’ quality of life, and different patients have different considerations in choosing treatment. In addition, sometimes the evidence in cancer care is inconclusive [15].

HLOC has not been closely studied in relation to the patient-oncologist discourse and decision making, although it plays an essential role in cancer-related medical decisions [8]. The study aims to trace HLOC aspects in the oncologic clinical encounter involving cancer patients through in-depth interviews with patients and family members. A qualitative approach offers an unprecedented opportunity to develop a deeper understanding of patients’ perceptions and HLOC characteristics. Learning from patients’ experiences in the clinical encounter may further support the development and implementation of models of oncologist-patient communication. While previous studies have examined the relationship between HLOC and decision-making using structured scales in quantitative methods, this study is unique in its in-depth insights into the patients’ inner world and perspective, using qualitative methodology.

## Materials and methods

The study was approved by the Ashkelon Academic College Ethics Committee (Approval #4–2019).

### Population sample and procedure

Semi-structured in-depth interviews were conducted with sixteen cancer patients and six family members during February-June 2020 after informed consent was obtained. Thirteen interviewees were female (eight patients and five family members), and nine were male (eight patients and one family member). The ages ranged from 37 to 73 among patients and from 24 to 72 among family members. Of the interviewees, only one patient and one relative were single; all the others were married. In terms of sacrifice, three family members were the spouses of a patient, one a daughter, one a granddaughter, and one a daughter-in-law. The interviewees suffered from different types of cancer (leukemia, lymphoma, melanoma, breast, bones, colon, and prostate) and were in various stages of the disease. The interviews lasted between 40 minutes and an hour. Ten interviews were conducted face-to-face in a place chosen by the interviewees (at a cafe near their home), and twelve were conducted over the telephone (due to coronavirus restrictions). The interviewees came from a wide geographical range spanning all the districts of Israel.

We developed a purposeful sampling, common to qualitative research. In the purposeful sampling, the interviewees were selected to obtain optimal variety and serve as potential sources of rich information to serve the study objectives. The informative richness and data saturation was achieved. To recruit interviewees, posts were posted in cancer patients’ forums on Facebook. Patients who were interested contacted the research assistant and were given a detailed explanation of the research purpose. Of the 27 people who applied, 22 were interviewed. Five potential interviewees regretted when we tried to make an appointment with them.

All interviews were conducted by a research assistant, a graduate student in clinical psychology. It was emphasized to all interviewees that their details would remain confidential, and their names would not appear in any published findings, and that they did not have to answer all the questions and could stop the interview at any point. In addition, all interviewees signed a consent form regarding the recording and transcription of the interview (S1 Appendix).

### Research tool

The in-depth interviews were semi-structured. The wording and order of the questions changed following the interview dynamics to maintain continuity and flow and encourage openness among the interviewees.

The topics for interviews included: personal details, social support, discussion with the oncologist about the treatment alternatives and implications, the responsibility of treatment choice, and who should decide on the medical treatment. For example: ‘Do you think the patient should decide on the medical treatment himself after receiving the information regarding the benefit and cost of the treatment from the doctor? Should the patient and the doctor share the decision? Should the physician decide for the patient based on their medical knowledge and familiarity with the patient?’ (S2 Appendix). The guide was developed in collaboration with two cancer patients and drew on our reviews of the literature. The guide was validated during a pilot interview. Through the pilot, we ensured a flow of the interview and an understanding of the questions. Family members were asked the same questions while adjusting. They had to describe the illness regarding the sick family member and voice their opinions regarding the rest.

### Data analysis

The interviews were transcribed and analyzed using a thematic analysis method in the ATLAS.ti v.8 software. The analysis included both deductive themes arising from the research topic and literature review on the HLOC model and inductive themes that emerged from the data [16]. The content of the interviews was analyzed in several stages according to Shkedi’s method [16]: initially, the focus was acquiring an in-depth and comprehensive knowledge of the data through a lateral reading of all the interviews. The next step was to identify ideas, categories, and themes related to the research questions. In the third stage, the characteristics and ideas were discussed while re-reading the transcripts to formulate the final themes.

The text analysis process involved three steps. In the first stage, two independent coders and medical sociologists determined whether the interview describes external or internal LOC processes with regard to medical decisions, including the direct question regarding SDM. For the level of reliability between the ranks according to the kappa index, Cohen’s kappa = 0.95. In the second stage, we defined two themes according to the HLOC model (external/internal LOC), eight sub-themes as emerged from the interviews, and a theme regarding SDM. In the third step, we re-read the interviews to encode them according to the themes and sub-themes. The reliability between the grades was high: Cohen’s kappa = 0.88.

Qualitative research, by its nature, requires a careful combination of external messages and the internal messages of the interviewees, so we must ensure the credibility and reliability of the research. According to Williams & Morrows’ approach [17], three main categories ensure credibility and reliability throughout the study. The first category is data reliability. As described above, the interviews were recorded and transcribed verbatim. The description of the findings was accompanied by citations from the interviewees and thus provided continuous evidence for matching the interpretation and the interviewees’ unique voices. Another category is a balance between the meaning given by the interviewees (subjectivity) and the researcher’s interpretation (reflectivity). The interviews were transcribed accurately by a professional, and the interpretive analysis was done close to the interviews. Finding common meanings in the analysis process and repetitive content reinforces credibility. The third category is a precise formulation of the research findings, possible ways of implementing them, as well as a reference to their meaning and implications in social reality, as described in the recommendations section.

## Results

### Participants’ characteristics

Participants’ characteristics and codification are available in Table 1.

**Table 1:** Clinical and social characteristics of the interviewees.

Code	Gender	Age	Marital Status	Cancer type
MP1	Man	55	Single	Lung cancer
MP2	Man	38	Single	Lymphoma
MP3	Man	37	Married+2	Leukemia
MP4	Man	64	Single	Lung cancer
MP5	Man	27	Single	Colorectal cancer stage IV
MP6	Man	30	Married+2	Pancreatic cancer
MP7	Man	70	Married+3	prostate cancer
MP8	Man	48	Married+2	Testicular cancer
FP1	Woman	46	Married+11	Stage IV lymphoma
FP2	Woman	40	Married	Melanoma
FP3	Woman	Na	Married+3	Stage III breast cancer, spread to the bones
FP4	Woman	64	Married+3	Breast cancer spread to the liver and bones
FP5	Woman	50	Married+3	Stage IV breast cancer spread to the bones
FP6	Woman	50	Devorce+3	Ovarian cancer
FP7	Woman	59	Married+3	Breast cancer
FP8	Woman	60	Married+4	Uterine cancer
Family Members:				
FFM1	Woman	50	Daughter in law	Her mother-in-law has stage IV lung cancer
FFM2	Woman	24	Granddaughter	Her grandma has lung cancer
FFM3	Woman	40	Daughter	Her father has lung cancer
FFM4	Woman	70	Wife	Her husband has lymphoma
FFM5	Woman	72	Widow	Her husband died of kidney cancer
MFM1	Man	52	Husband	His wife has a cancerous tumor in her throat

### Themes

Following the HLOC model, the findings were divided into external and internal LOC themes. In addition, we added a theme regarding SDM. Table 2 presents themes and sub-themes that emerged from the data.

**Table 2:** Themes and sub-themes.

Themes	Sub-themes
Internal LOC	Asking for a second opinion
	Choosing the treatment themselves, negotiating with the doctor
	Actively discussing issues with the doctor and asking questions
	Looking for information and reading every relevant new study published
	Fighting for their rights
External LOC	Accepting solely oncologist recommendations
	Lean on powerful others (e.g., rabbi, family member)
	Believe in fate
Active vs. Passive decision making (direct question)	

### Theme 1-internal LOC

#### Asking for a second opinion

Half the respondents shared that they had sought a second opinion. Two were referred by their oncologist, who was deliberating further treatment, and the rest sought a second opinion at their own initiative, to be sure that the treatment proposed to them, or their ill family member was indeed optimal, mainly when it entailed surgery: "There were very difficult deliberations because it is life-threatening from every angle. Truly a question of life and death. The doctor told me: This is the time for you to consult with other doctors." When this patient was invited to participate in an experimental study, she relayed, "We really deliberated; we consulted with another professor and doctors abroad, and everyone thought that this was the direction to take" (Female patient 1 –FP1).

Another patient shared: "The oncologist decided on 16 chemotherapy treatments and that’s it. But I had something to say because I did not stop at one doctor. Look, it’s not easy to tell an oncologist, ’Look, I consulted with other doctors,’ because what, you don’t trust me? But from the beginning I put it on the table" (FP7).

#### Choosing the treatment themselves, negotiating with the doctor

A third of the patients chose the treatment themselves: "The responsibility for choosing the treatment falls on me, with the doctor ensuring that I am well aware of what I’m getting into. I was offered alternatives and told, ’Choose what suits you.‴ (FP8). One family member relayed that his ill wife chose her own treatment: "We were offered treatments, and she chose. It is beyond me to choose for her. She must decide everything herself" (Male family member 1 –MFM1). Some patients negotiate with the doctor: "I started with a breast surgeon who did a mastectomy, then I went to oncology, and we had a discussion. For example, I wanted longer intervals in chemotherapy. It’s usually two weeks; I asked for every three [weeks]. Same with the type of treatment and radiation and amount, and ultimately the oncologist accepted it" (FP3).

#### Actively discussing issues with the doctor and asking questions

During their illness, some patients become empowered and learn how to conduct a serious discussion with their doctor regarding future measures, ask questions, and even request studies that validate the doctors’ recommendations. They did not want to be passive in the decision-making. For example, interviewee FP7 stated: "One of the important things is that I learned to ask questions. I learned that the doctor is not God. I listen to the doctors’ answers, and if they do not satisfy me, I ask more questions, and if it does not suit me, I consult with other doctors." Patients’ family members underwent a similar process: "I am someone who asks questions, my husband, for example, it doesn’t interest him. He doesn’t ask. The one who asks questions is me, and I always get a response" (Female family member 4 –FFM4). Likewise, interviewee FFM5 shared: "The doctors suffered a lot because of my knowledge and my big mouth. Because I did not give up. I made things very hard for them."

The study yielded an interesting finding on gender roles. According to gender-related stereotypes, women are usually expected to be passive, subservient, and non-dominating. Men should be independent, eschewing weakness and emotion [18]. But the interviews revealed the opposite. Men turned out to be more passive, trusting the doctor without asking too many questions. Women appeared to be more assertive, making decisions and h independently having an internal LOC higher than that of men. We assume that women feel a greater need to recover because they are afraid of abandoning their children and family, so their internal engine to survive is stronger than men’s. Indeed, women cancer patients display better survival rates than men [19].

#### Looking for information and reading every relevant new study published

These patients ask the doctor for information about innovative technologies but do not stop there. They are constantly seeking material themselves. They are "researcher patients." Interviewee FP3, for example, stated: "Every step is explained to me, every new drug being researched. I read everything. I know that if my drug stops working, the next drug is X, and then comes drug Y. That means that for the entire course of what we are doing, I have a plan going ahead." Interviewee FP4, although she reads and researches for herself, filters out certain things for her own sake: "I read about every drug I’m given. I read everything I could. But there are some things I intentionally avoid so as not to lower my spirits because I need to ’carry on’ and have strength."

Family members of patients also read and research: "I started searching for a suitable drug, and when we met with the doctor, he said it was a possibility and that he had patients whom it helped a lot" (1FFM). Interviewee FFM2 added: "We read a lot by ourselves online. We conducted very comprehensive research and searched on our own for drugs that might be suitable."

#### Fighting for their rights

One of the significant indicators of internal HLOC is the fight for one’s rights and insistence on ultimately getting the best care even if it is very expensive for the sick fund or insurance company. Patients had less energy to fight for themselves, although they did voice anger and frustration over the bureaucracy involved in receiving authorizations and compensation for tests and drugs not covered by the national healthcare system: "What’s happening is shameful and disgraceful. When the doctor decided to give me a drug that is not covered by the healthcare system, after trying all the options, we approached our Health maintenance organization (HMO). The HMO absolutely refused to fund it because according to them I was missing an MSI test (Micro Satellite Instability test), which in my case cannot be conducted. As far as they are concerned, we should pay for three treatments, at $23,000, and then they will consider it."

This is where family members mobilize, and all recounted how they had to fight for their ill family member. For example: "In the end, I despaired and physically went to the main office of the health network, to the director-general, and after a twenty-minute argument, we got the authorization. Everything has to be taken by force. And if people don’t know how to fight… I knew I called every day, I went to the management, and how many people don’t do that?" (FFM1). Interviewee MFM1 described how he fought for his ill wife even when it came to hospital tests: "If you don’t know what your rights are, you will not automatically get them. And if let’s say, she needs a test, I bring her to the place for the test, and they tell me two more months. What two months? The oncologist says the test should have been done yesterday. I brought her by wheelchair, positioned her behind the doorway, and said hello, we came for a test. They say: Do you have an appointment? I say no. So they asked, what should we do with those who have an appointment? I said send them home. What are you such a hero for? I said I am not a hero, but my wife’s life is important to me. After some arguments, she had the test that day. I fight at such a level that the oncologists ask me jokingly: If we need a test, can you help us arrange an appointment?"

### Theme 2-external LOC

#### Accepting solely oncologists’ recommendations

Half the patients said they trust their oncologist implicitly, that they do not argue or question the oncologist’s assertions and treatment recommendations. For example: "The doctor said so, and I said amen" (MP7); "The doctor said so, you cannot say no. What he decides is the best" (MP5); "I gave blanket approval for anything the doctor asked (FP1)."

These patients trust the oncologists treating them, particularly when the oncologists are well regarded in their field. They do not even look into other treatment alternatives or seek a second opinion: "My doctor is a great authority; I never discussed alternative treatments with her. What she said seemed right and accurate to me" (FP5); "My oncologist is considered eminent, and I accepted what he proposed. Nor do I have the strength to conduct research and assessments to look for a different doctor" (MP7); "Some people engage in research and can tell the doctor which treatment they want… But in my view, doctors have cumulative experience from other patients. It is knowledge acquired over the years. So I presume they know what they are doing; therefore, it’s unnecessary" (MP4).

Interviewee MFM1, husband of a cancer patient, tried to explain the need to trust the doctor: "If you don’t trust the doctor, don’t go to him. I hear family members of patients getting angry and saying to the doctor, ’why this, why that?’ If you know better, take the patient home, take care of him, and do whatever you want. But if you come here, let them provide treatment as they understand it."

#### Lean on powerful others

Patient FP6 describes how family members managed her and, in consultation with the doctor, decided on her treatment: "My partner’s sister is a nurse, so they made the medical decisions and managed me. I chose what they said. They spoke with the doctor: he said surgery, so surgery. I did not want chemotherapy, but he said to do it, so I did." Even when it came to a study, an idea she initially opposed, the family compelled her to participate: "At first, I objected very, very much; I did not want a study; I did not want to take any pill that was only at the experimental stage. The family pressed very strongly and pretty much compelled me to participate."

Patient FP1 went to a very well-known rabbi to resolve a treatment dilemma: "There was a stage at which the doctor said he was very undecided and at the gut level favored a transplant. I said that my gut feeling was not in favor. Ultimately, I consulted with Rabbi Kanievsky, who said not to have the transplant, and that is what decided it for me, and I didn’t do it."

#### Believe in fate

Some patients do not investigate or ask questions, in part because they believe in fate and luck: "I believe in fate; ultimately everybody ends up at the cemetery" (MP1). Interviewee FP8 related, "Everyone has their own luck. Some people live for two years after surgery, then die. Some for five years. There is someone elderly who had the surgery and is still alive eleven years later. It’s all a matter of luck." Interviewee FP6 spoke about the importance of trust and belief in the success of treatment: "My understanding is that if you fight the doctors and proceed out of resistance rather than trust and faith, the treatment could be unsuccessful."

### Theme 3-active vs. passive decision making

HLOC, whether external or internal, affects patients’ perspectives regarding their desire or need to participate in medical decision-making. We explicitly asked who should decide on treatment—the doctor, the patient, or both jointly. Responses were evenly distributed among the options: only the doctor (n = 8), jointly (n = 7), only the patient (n = 7).

The stated reasons why *the doctor* should make the decision usually referred to the patent’s lack of knowledge versus the doctor’s knowledge: "Most people do not have enough knowledge about their case. What are they capable of deciding? What do I know about radiation? Any such territory is a world onto itself" (MP7); or they had total faith in the doctor’s expertise: "I think the doctor should decide. Maybe because I happened to get a doctor who is really Number 1, so I never thought I needed to decide anything jointly with her. What she said, she said. I never argued or questioned" (FP5).

Those who support *shared decision-making* want the doctor to lay out all the options, outcomes, and side effects, and want the discussion of alternatives to take place "at eye level," mainly because it is the patient who physically experiences the treatment: "The decision should be made jointly. It is a must. Because in undergoing the treatment, the patient should be certain that what the doctor is doing is first of all by consent and with awareness of every upcoming step. These are not easy steps. Chemotherapy treatments are very, very hard" (FP3). Interviewee FP8 added: "A joint decision with the doctors. The patient does not have the information, knowledge, experience that the oncologist does. I have friends who sought a second opinion, returned to the oncologist, consulted again together, and reached a joint decision. The oncologist does not have to be accepted as [someone to] ’observe and sanctify.’ It is therefore essential that the decision be made jointly." Interviewee FFM2, a patient’s granddaughter, concurred: "It should be a joint decision. Not everyone truly understands all the repercussions of every treatment, and the doctor should not put words in the patient’s mouth. He should patiently explain where each option might lead, and then they decide together."

Contrastingly, some believe that the decision should be made solely by the patient, after acquiring knowledge about the disease and understanding the repercussions of each treatment: "At first, when the patient does not really know much about the disease, the doctor should be more dominant in decision-making. But in later stages, when the patient understands much more, then the doctors’ role is to explain the side effects and chances, but the final decision should be made by the patient" (MP2). Moreover, patients should be sure it accords with their lifestyle: "The patient should take into account all the information and consider whether the treatment suits his lifestyle and day-to-day life, then decide" (FFM3). Patient FP2 summarized: "The doctor should offer all the options, but we choose. It’s a personal choice."

## Discussion

The study aimed to trace the dominant LOC in the cancer patient-oncologist encounter. Previous studies have examined it using the self-report Multidimensional HLOC scale [6, 20–22]. In the present study, however, we have taken a qualitative approach to gain an in-depth understanding of cancer patients and family members’ experiences.

Internal LOC refers to the extent to which individuals believe in their own ability to influence their health [7]. Cancer is in many cases a chronic, sometimes terminal, disease that requires very severe treatments with side effects that impair patients’ quality of life. It was therefore expected that internal LOC would be more dominant than external LOC, and that patients would prefer to take responsibility for their health and quality of life. Despite this, we found that the dominant approach for most of them was external LOC (six men, three women, and one family member). Two women and four family members had a dominant internal LOC dimension, and the rest demonstrated a combined internal and external LOC (two men, three women, and one family member). Similarly, a study by Lin and Tsay [23] found that more cancer patients had a powerful others HLOC rather than an internal HLOC.

Some interviewees stated that they trust their oncologist and believe they must do what the oncologist say without arguing or seeking information on the disease and different treatment options. On the one hand, some cancer-related decisions can rest with the recommendation of the oncologist and may not need or benefit from a second opinion or patient “research.” In this context, Wallston and Wallston [24] explained that doctors are perceived as authority figures, and it is logical to assume that people endorsing powerful others HLOC attitudes would respect and trust the oncologist with their "eyes shut." The idea that individuals who endorse beliefs that health professionals control their health are likely to have feelings of trust toward physicians is consistent with Brincks et al. [8] On the other hand, this kind of passive behavior can impact on low internal HLOC, especially when suffering from a disease that can be terminal, and new treatment technologies are constantly evolving. Arraras et al. [3] indicated that low internal HLOC is related to distress, allowing for a passive and avoidant coping style. Internal HLOC is essential in managing the cognitive perception of the threat of illness, while external LOC may be beneficial until it reaches a point of creating dependency [25].

SDM is one of the essential dimensions in a patient-centered care approach and an ethical framework for decision-making in cancer care [26]. SDM is based on the available evidence, along with the patient’s values, wishes, and preferences [27]. Several barriers can prevent cancer patients from participating in medical decisions: insufficient knowledge, lack of experience, reduced mental capacity, and inadequate resources [28]. The interviewees raised these points but did not criticize the doctor’s policy; on the contrary, they accepted it. Interviewees who did not support active decision-making said they do not have enough understanding and are not up to date on current studies, so the doctor should decide, as he is experienced and has treated many patients. Rocque et al. [29] described three best practices for implementing SDM: (1) engagement of stakeholders who have an interest in SDM, (2) development of an evidence-based SDM tool, and (3) development of infrastructure needed for encouraging patient engagement in decision-making. We think that another essential practice should be to ask the patient whether he wants SDM. Our interviewees were divided on this issue, ranging from an exclusive decision of the oncologist to a joint decision to an exclusive decision of the patient. Therefore, it cannot be assumed that all patients are interested in SDM. In addition, some patients described a dynamic process whereby the focus shifts from external to internal or to more shared decision-making; thus, a more active and nuanced perspective became apparent. In this context, the level of health literacy of the patients should also be considered. Are they able to search, read and understand studies or not?

When comparing the sub-themes of internal LOC, as they emerged from the interviews with the items in the HLOC scale (e.g., I am directly responsible for my health getting better or worse; Whatever goes wrong with my health is my own fault), a unique contribution of this paper becomes evident: it seems that the scale can be adapted and improved by using specific items/measures for cancer patients that arose from their own experience of struggling with the disease.

### Study limitation

Interviews were conducted only in Israel, which has a public health system. Furthermore, specific cultural aspects may play a role in the responses. In countries with a different healthcare system, the type and level of LOC may differ. In addition, the sample was relatively small. However, given the qualitative approach, we stopped interviewing when we observed that no additional subjects were mentioned. Moreover, social desirability bias may also be present, as patients may selectively share perceptions they view as more acceptable or socially desirable.

### Implications

The findings reflect the need to devote comprehensive attention to cancer patients’ perceptions and experiences in the clinical encounter. A patient-centered care approach and a personalized framework for oncologist-patient discourse in cancer care are essential to achieving better treatment outcomes. The current HLOC tool [30] includes five dimensions: Internal, Chance, Doctor, Powerful others, and God. Based on the exploratory findings of this study, further examination, development, and validation of an up-to-date HLOC questionnaire for cancer patients is recommended. Mainly including items describing shared decision making and adapting internal LOC aspects to the cancer disease, e.g., asking for a second opinion, and actively discussing issues with the doctor.

## Conclusions

This qualitative study provides insight into aspects of LOC in the clinical encounter involving cancer patients. A considerable proportion of patients and caregivers still perceive doctors as ultimate authority figures. Accordingly, the dominant approach of most participants was external LOC, although previous studies ascribed this to an avoidant coping style. The findings revealed sub-themes of internal LOC that could be implemented in the content-related adaption of the HLOC scale to understand better the characteristics of LOC and its implications on SDM among cancer patients.

## Supporting information

S1 Appendix Participant consent form. (DOCX)

S2 Appendix In-depth interview guide. (DOCX)

## Abbreviations

FFM: Female family member
FP: Female patient
HLOC: Health locus of control
HMO: Health maintenance organization
LOC: Locus of control
MFM: Male family member
MP: Male patient
MSI test: Micro Satellite Instability test
SDM: Shared decision-making
