# Supplementary material for: Health locus of control in cancer patient and oncologist decision-making: An exploratory qualitative study
Source: PLoS One. 2022 Jan 27;17(1):e0263086. doi: 10.1371/journal.pone.0263086 (PMC8794183; doi:10.1371/journal.pone.0263086)
Supplement: S1 Appendix — (DOCX) [file pone.0263086.s001.docx]

**S1. Appendix - Participant Consent Form**

I, the below signed:

Last name: ______________________ First name: __________________________

1. Hereby declare that I am a competent adult and agree to participate in the study described in this document.
2. Confirm that ____________________ explained to me that the current study on “**Patient-oncologist clinical encounter: in-depth interviews with cancer patients”** was approved by the Ethics Committee of Ashkelon Academic College, and I am aware of the following:
   1. That I am free to withdraw from the interview at any time.
   2. That my personal identity will remain fully confidential in scientific publications or any other publication of this study.
   3. Should any problem arise related to the study, I can turn to the researcher for additional consultation.
   4. I am not aware of any medical, legal or other impediment to my participation in the study.
3. Hereby declare that I was provided with detailed information on the study, including information on the study objectives, methodology, anticipated duration, risks and discomfort that might arise owing to participation in the study.
4. Hereby declare that my consent was given voluntarily after I read and understood all the above.
5. I give my permission to record and transcribe my comments.

| Name of participant | Signature | Date |
| --- | --- | --- |

Researcher’s declaration

I have received the above consent, after I explained all of the above to the participant and verified that s/he understood all of the explanations.

| Name | Signature | Date |
| --- | --- | --- |
