# Supplementary material for: Health locus of control in cancer patient and oncologist decision-making: An exploratory qualitative study
Source: PLoS One. 2022 Jan 27;17(1):e0263086. doi: 10.1371/journal.pone.0263086 (PMC8794183; doi:10.1371/journal.pone.0263086)
Supplement: S2 Appendix — (DOCX) [file pone.0263086.s002.docx]

**S2. Appendix - In-Depth Interview Guide**

1. Tell me a little about yourself, when the disease was discovered, and how it affected you emotionally / family / financially / in everyday life?
2. Do you have any support from the family or another close person while dealing with cancer when you arrive for treatments, at-home care, making decisions in dilemmas related to treatment?
3. Is there a discussion with your oncologist about the treatment alternatives?
4. Has your oncologist offered you innovative treatments that are not in the health basket?
5. How did you choose your preferred treatment? Was the economic consideration the overriding consideration or the clinical consideration?
6. Does your oncologist discuss with you the meanings of the clinical studies on which he relied when he offered innovative treatment (concepts such as time without disease progression, median life expectancy, etc.)?
7. Has your oncologist transferred to you the responsibility to choose the type of treatment?
8. Would you like to receive more information from your oncologist regarding the treatment implications for the family members around you?
9. Does the attending physician discuss with you the treatment implications, your emotional state, and how the treatment will affect your quality of life?
10. Do you think the patient should decide on the medical treatment himself after receiving the information regarding the benefit and cost of the treatment from the doctor? Should the patient and the doctor share the decision? Should the physician decide for the patient based on their medical knowledge and familiarity with the patient?
